# Crosstalk between H9N2 avian influenza virus and crypt-derived intestinal organoids

**DOI:** 10.1186/s13567-017-0478-6

**Published:** 2017-11-02

**Authors:** Lulu Huang, Qihang Hou, Lulu Ye, Qian Yang, Qinghua Yu

**Affiliations:** College of Veterinary Medicine, Nanjing Agricultural University, Weigang 1, Nanjing, 210095 Jiangsu China

## Abstract

The spread of Avian influenza virus via animal feces makes the virus difficult to prevent, which causes great threat to human health. Therefore, it is imperative to understand the survival and invasion mechanism of H9N2 virus in the intestinal mucosa. In this study, we used mouse threedimensional intestinal organoids that contained intestinal crypts and villi differentiated from intestinal stem cells to explore interactions between H9N2 avian influenza virus and the intestinal mucosa. The HA, NA, NP and PB1 genes of H9N2 viruses could be detected in intestinal organoids at 1 h, and reached peak levels at 48 h post-infection. Moreover, the HA and NP proteins of H9N2 virus could also be detected in organoids via immunofluorescence. Virus invasion caused damage to intestinal organoids with reduced mRNA transcript expression of Wnt3, Dll1 and Dll4. The abnormal growth of intestinal organoids may be attributed to the loss of Paneth cells, as indicated by the low mRNA transcript levels of lyz1 and defcr1. This present study demonstrates that H9N2 virus could invade intestinal organoids and then cause damage, as well as affect intestinal stem cell proliferation and differentiation, promoting the loss of Paneth cells.

## Introduction

In China, low pathogenicity avian influenza (LPAI) viruses of the H9N2 subtype have become endemic. Notably, H9N2 virus has been detected in multiple avian species, including chicken, duck, quail, pheasant, partridge, pigeon, silky chicken, chukar, and egret, which has resulted in significant economic losses [1, 2]. H9N2 viruses have undergone extensive reassortment with many subtypes of AI viruses, including HPAI, H5N1, and H7N3 viruses; moreover, the H9N2 virus poses a significant zoonotic threat [3]. H9N2 viruses have also been well known to donate internal genes to the highly pathogenic H5N1 avian influenza viruses in humans in Hong Kong [4].

Avian influenza virus (AIV) mainly infects through the respiratory tract, resulting in severe respiratory syndrome or even death. However, the H9N2 virus can also replicate in avian guts and spread by fecal–oral transmission [5]. With the annual migration of birds, H9N2 virus can spread along migration routes, which makes it hard to prevent and control. Previous studies have established that AIV can invade intestinal cells, such as HT-29 and Caco-2 cells, and cause severe epithelial apoptosis [5, 6]. However, the intestinal mucosa contains intestinal crypt and villi that can be periodically replaced by intestinal stem cells (ISC) in the crypt. In small crypt base columnar (CBC) cells, which are intermingled with Paneth cells, Barker et al. have shown that Lgr5^+^ CBC cells possess intestinal stem cell properties: long-term self-renewal and multipotential differentiation [7]. Moreover, the mucosa contains goblet cells and Paneth cells that can secrete antimicrobial proteins. To date, the use of single cells to explore cross-talk between pathogenic micro-organisms and the host is not accurate or reliable.

A major breakthrough was made by Dr Hans Clevers et al. who for the first time showed that intestinal stem cells can differentiate into all intestinal epithelial cell types (i.e., enterocytes, Paneth cells, Goblet cells, enteroendocrine cells, as well as stem and progenitor cells) using “mini-gut” or “organoid” systems [8–10]. Intestinal organoids are three-dimensional structures of cultured intestinal cells that incorporate many key features of the intestinal epithelium in vivo, including a crypt-villus structure that surrounds a functional central lumen, and provides a convenient and physiologically relevant model for studies of intestinal biology.

To date, limited data are available that describe virus invasion into intestinal organoids, and the influence of viruses on intestinal stem cells. Here, we assessed whether H9N2 virus could invade mouse intestinal organoids and we assessed the effects of virus infection of intestinal stem cells and Paneth cells.

## Materials and methods

### Reagents and antibodies

Advanced DMEM/F12 medium, N2 supplement, and B27 supplement were purchased from Invitrogen (Grand Island, NY, USA). Recombinant EGF, Noggin and R-spondin were obtained from Peprotech (Rocky Hill, NJ, USA) and were added to advanced DMEM/F12 medium to form ENR-DMEM medium. Anti-influenza virus HA protein and anti-influenza virus nucleoprotein antibody-FITC were purchased from Abcam (Cambridge, MA, USA).

### Viruses and animals

Influenza virus (A/Duck/NanJing/01/1000 [H9N2]) was generously supplied by the Jiangsu Academy of Agricultural Sciences (Nanjing China) [11]. C57BL/6 mice (6 weeks old, specific-pathogen-free [SPF]) were purchased from the Animal Research Centre of Yangzhou University. This study was approved by the Ethics Committee for Animal Experimentation of the Nanjing Agricultural University. All animal care and use procedures were conducted in strict accordance with the Animal Research Committee guidelines of the College of Veterinary Medicine at Nanjing Agricultural University.

### Establishment of an intestinal crypt culture system

Intestinal crypts were isolated from C57BL/6 mouse, and intestinal organoids were established and cultured as described previously [8]. Briefly, crypts were released from mouse small intestine tissues by incubation for 30 min at 4 °C in DPBS that contained 2 mM EDTA. A total of 10 μL of crypts were mixed with 50 μL of Matrigel (BD Bioscience, San Jose, CA, USA) and plated in 24-well plates. After the polymerization of Matrigel, complete crypt culture medium that contained advanced DMEM/F12 supplemented with 2 mM GlutaMax (Life Technologies, NY, USA), 10 mM HEPES, 100 μg/mL penicillin/streptomycin, N2 supplement, B27 supplement and growth factors [50 ng/mL EGF, 100 ng/mL Noggin, 500 ng/mL R-spondin, and 10 μM Y-27632 (Selleck, Munich, Germany)] were added.

### H9N2 virus infection of cultured mouse intestinal organoids

To determine whether H9N2 could invade intestinal organoids, organoids were co-cultured with H9N2 virus. Firstly, intestinal organoids were removed from the Matrigel in which they were embedded and co-cultured with 6 μL H9N2 (10^6^ EID_50_) virus for 1 h in 1.5-mL Eppendorf tubes. Then, the medium was removed, and complete crypt culture medium supplemented with 2 μg/mL TPCK-trypsin. Intestinal organoids were collected at 1, 12, and 48 h post-infection (hpi) for qRT-PCR for assessments of mRNA transcript expression of AIV (HA, NA, NP and PB1), and morphological changes were observed by a confocal laser fluorescence microscope (Zeiss 710; Carl Zeiss, Oberchoken, Germany).

Total RNA from intestinal organoids was extracted by RNAios Plus (Takara, Dalian, China). Reverse transcription of RNA was performed with customized primers (Table 1) designed to amplify fragments of the target genes. In this experiment, the thermal cycling conditions were 5 min at 95 °C, followed by 40 cycles of 15 s at 95 °C and 31 s at 60 °C using an Applied Biosystems 7500 Real-Time PCR system. Fold-increases in the mRNA levels of target genes were calculated using the 2^−ΔΔ^ CT method, normalizing expression levels to those of GAPDH.

**Table 1:** **Primer sequences for RT-qPCR**

Target genes	Primer sense (5′–3′)	Primer antisense (5′–3′)	Product size (bp)
HA	AGACCATCGGCTGTTAATGG	TTGTGTATTGGGCGTCTTGA	235
NA	TTCAGGCAGAATGAATGCAG	TGCGGAAAGCCTAATTGAGT	213
NP	GAAATCCTGGGAATGCTGAA	AACACCTGGCTGTTTTGGAG	202
PB1	AGCGGGTATGCACAAACAGA	ATAAGTCTGGCGACCTTGGG	150
mLyz1	GCCAAGGTCTACAATCGTTGTGAGTTG	CAGTCAGCCAGCTTGACACCACG	290
mDefa6	CCTTCCAGGTCCAGGCTGAT	TGAGAAGTGGTCATCAGGCAC	317
mDefcr1	TCAAGAGGCTGCAAAGGAAGAGAAC	TGGTCTCCATGTTCAGCGACAGC	280
mWnt3	CTCGCTGGCTACCCAATTTG	CTTCACACCTTCTGCTACGCT	165
mAxin2	TGACTCTCCTTCCAGATCCCA	TGCCCACACTAGGCTGACA	105
mGAPDH	ATGGTGAAGGTCGGTGTGAA	TGGAAGATGGTGATGGGCTT	217
mLgr5	CTGAGACAGGTTCCGGAGGA	GAGATGCAGAACCACGAGGC	276
mBmi1	CAAGCTGCCCGTCTATACCC	AGAGGGGTAGGTGGCAAAGA	199
mDll1	CAGGACCTTCTTTCGCGTATG	AAGGGGAATCGGATGGGGTT	168
mDll4	TTCCAGGCAACCTTCTCCGA	ACTGCCGCTATTCTTGTCCC	102

At 48 hpi, intestinal organoids were fixed with 4% paraformaldehyde in PBS for 1 h at 4 °C and were then permeabilized with 0.4% Triton X-100 for 30 min. After staining with HA (red) or NP (green) and DAPI (blue), organoids were observed by confocal microscopy.

### Detection of cellular proliferation and apoptosis after infection in intestinal organoids

Proliferation in intestinal organoids was measured by EdU (5-ethynyl-2′-deoxyuridine) incorporation after H9N2 virus infection. In brief, EdU (1:2000) was pre-incubated in ENR-medium for 2 h. The nuclei were stained by Hoechst33342. Cells were then detected with a fluorescence microscope.

Apoptotic cells were detected by Annexin V and propidium iodide (PI) staining assay (Miltenyi Biotec, Shanghai, China) according to the manufacturer’s protocols. Briefly, organoids were digested with accutase (Millipore) for 25 min at 37 °C and single-cell suspensions were obtained. Cells were harvested and washed with PBS. Following this, the cells were incubated with 5 μL Annexin V-FITC and 5 μL PI-FL3 for 10 min and detected with FACS Calibur (Becton, Dickinson and Company, USA). Single cell was gated using FSC and SSC parameters and apoptotic cells of organoids were analyzed at FL-1 and FL-3 by FACS.

### Paneth cell variation after invasion of H9N2 virus

According to a previously reported method, RT-qPCR analysis of maker genes (Lyz1, defcr1 and defa6) expression levels in Paneth cells were tested at 1, 12 and 48 hpi [12, 13].

### Effects of H9N2 virus infection of stem cells

Intestinal organoids were grown on 24-well plates and then co-cultured with H9N2 virus for different time points. RT-qPCR analysis was used to assess mRNA transcript levels of maker genes for stem cells (Lgr5 and Bmi1) and components of the Wnt signal pathway (Wnt3, Axin2) and Notch signal pathway related genes (Dll1, Dll4) at 1, 12 and 48 hpi.

## Results

### Assessment of the growth of mouse intestinal organoids

**Figure 1:**
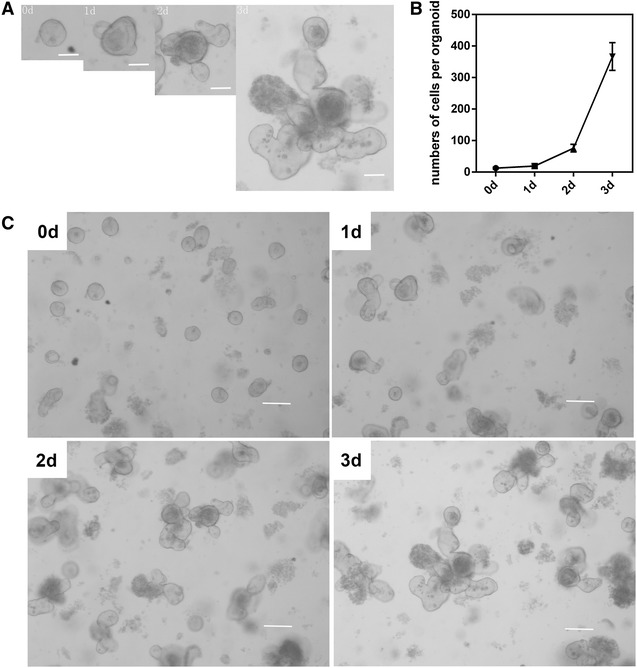
**Establishment of an intestinal crypt culture system.** Crypts from mouse small intestines were seeded into Matrigel and cultured for 72 h to obtain well-developed organoids. **A** Kinetics of the growth of a single isolated crypt. **B** Cell counts of a single organoid cultured for 4 days. **C** Single isolated crypts efficiently form large crypt organoids within 4 days, along with several surrounding crypt-like domains. Mean values ± SE of triplicate assays are shown. Bars: 20 μm (**A**); 50 μm (**C**).

Intestinal crypts were isolated from the mouse jejunum and cultured in ENR medium to form intestinal organoids, which contains all kinds of epithelial cells, such as absorptive cells, Paneth cells, goblet cells and so on. On the first day post-isolation, the organoids were rounded and bright, then organoids began to bud on the second day, and more budding crypts became visible by the third day (Figures 1A and C). The cell number per organoid also increased over time (Figure 1B).

### Invasion of H9N2 virus into mouse intestinal organoids

**Figure 2:**
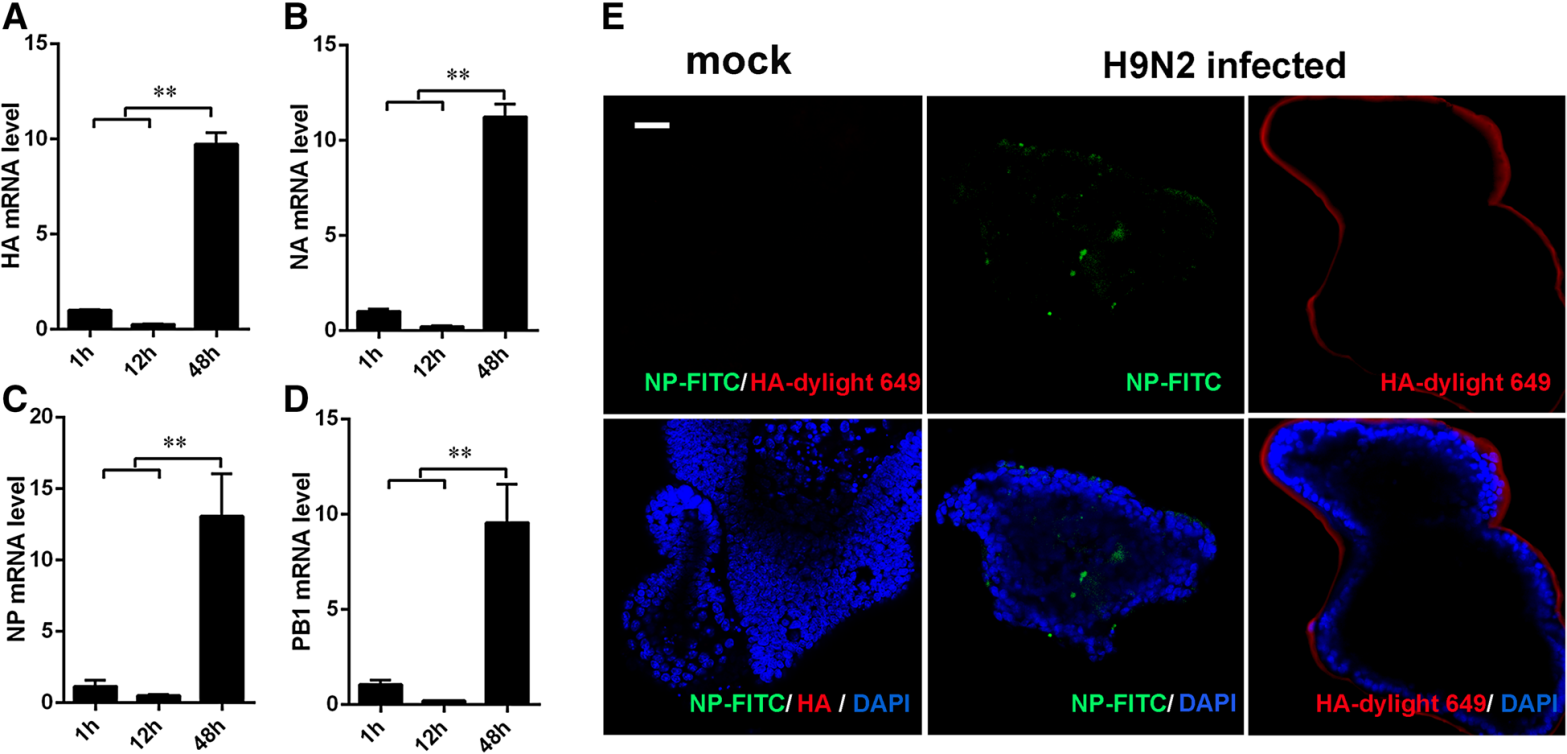
**AIV replicates in mouse intestinal organoids.** Replication of H9N2 virus (10^6^ EID_50_) in mouse intestinal organoids. **A**–**D** GAPDH-normalized mRNA transcript levels of H9N2 HA, NA, NP and PB1 RNA are shown as fold-increases relative to those at 1 h post-infection. **E** Virus was evaluated by immunofluorescence staining for nonstructural protein NP (green) and structural protein HA (red), and DAPI (4,6-diamidino-2-phenylindole) to stain the nucleus (blue). Mean values ± SE of triplicate assays are shown; *P < 0.05; **P < 0.01. Experiments were performed in triplicate. Bars: 25 μm (**E**).

Invasion of H9N2 virus is the basis for virus replication, then causing damage to host cells. The mRNA transcripts of virus proteins, such as HA and NA, were detected in the first hour post-infection, and the mRNA transcript levels became reduced by 12 h. However, the expression levels of HA (Figure 2A) and NA (Figure 2B) markedly increased over the 48 h period post-infection. Similar patterns of NP (Figure 2C) and PB1 (Figure 2D) expression were also detected. Furthermore, H9N2 virus invasion was confirmed by confocal microscopy. HA protein of the virus labeled with dylight 649 was clearly visible around the organoids (Figure 2E). Moreover, labeled NP was also detected inside of the mouse intestinal organoids (Figure 2E).

### Morphological changes of organoids after H9N2 virus invasion

**Figure 3:**
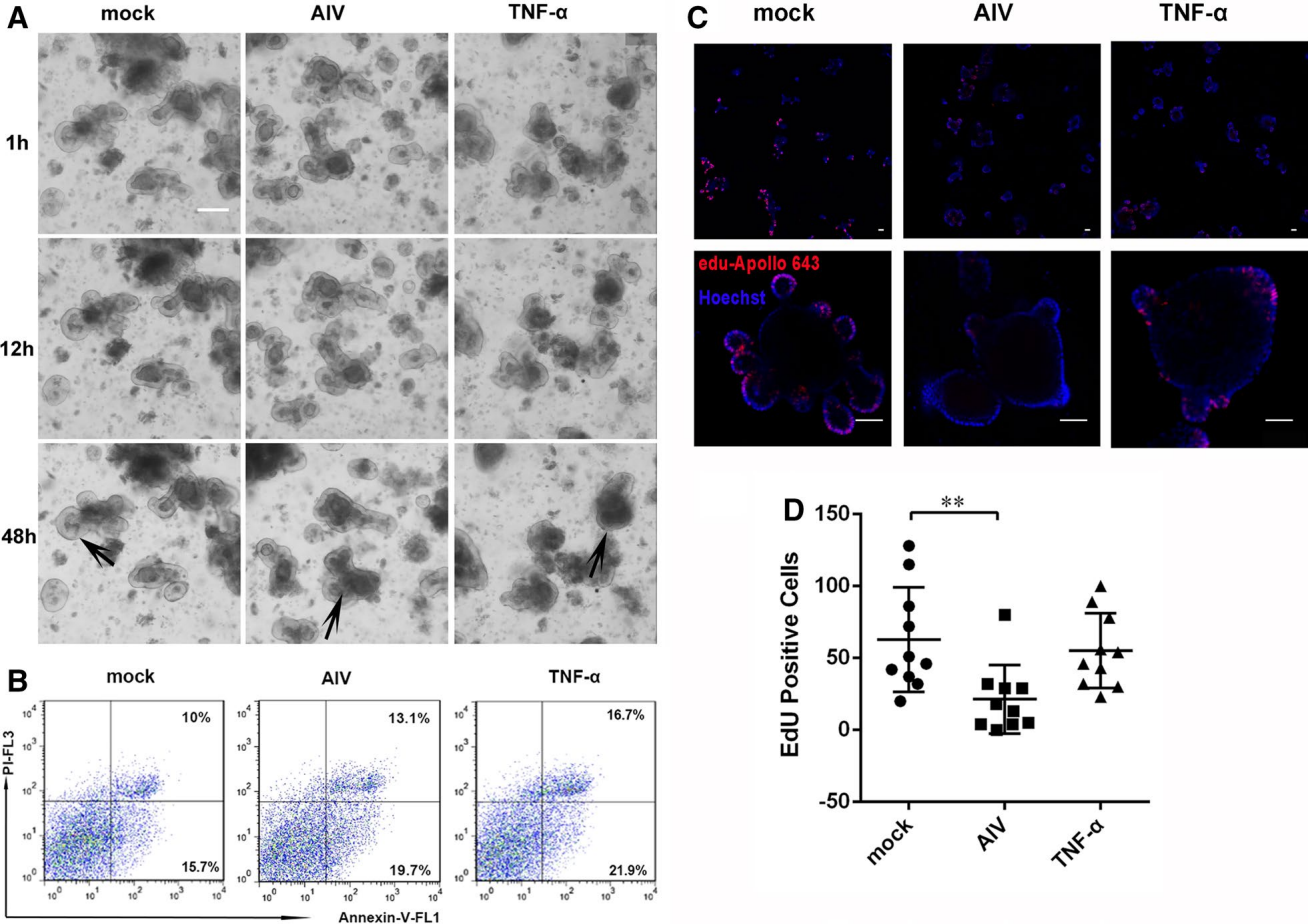
**Changes in organoid morphology after infection with H9N2 virus.** Intestinal organoids from mouse small intestines were seeded into Matrigel and cultured for 72 h to obtain well-developed organoids. Organoids were treated with medium, H9N2 virus, or TNF-α (100 ng/mL). **A** Crypt morphology was assessed by light microscopy. **B** Annexin V–PI double staining was performed to differentiate cells in early apoptosis (Annexin V + , PI −) from those in late apoptosis (Annexin V + , PI +). **C**, **D** EdU incorporation (2 h) in organoids cultured in the presence of intestinal organoids. The results are representative of data from three independent experiments. Each data bar shown represents mean values ± SE of triplicate assays. *P < 0.05; **P < 0.01. Bars: 20 μm (**A**). Bars: 50 μm (**C**).

The intact morphology is the guarantee of the normal function of intestinal organoids. TNF-α causes damage to the intestinal epithelium and thereby contributes to the pathogenesis of various inflammatory disorders of the intestine [14, 15]. In order to explore the effect of H9N2 virus invasion of intestinal organoids, TNF-α (tumor necrosis factor-α) was used as a positive control. Intestinal organoids treated with H9N2 virus and TNF-α were clearly visible with intact villi and crypts. However, the organoids were damaged and deformed at 12 h post-TNF-α treatment. Moreover, H9N2 virus invasion also caused damage to the intestinal organoids at 48 hpi, with atrophic villi and crypts (Figure 3A). At the same time, Apoptotic cells was detected by Annexin V and propidium iodide staining assay. After H9N2 virus infection for 48 h, the number of apoptosis cells was increased significantly by FACS (Figure 3B), which is consistent with reduced proliferation cells staining with EdU (Figures 3C and D).

### Virus infection results in the loss of Paneth cell function

**Figure 4:**
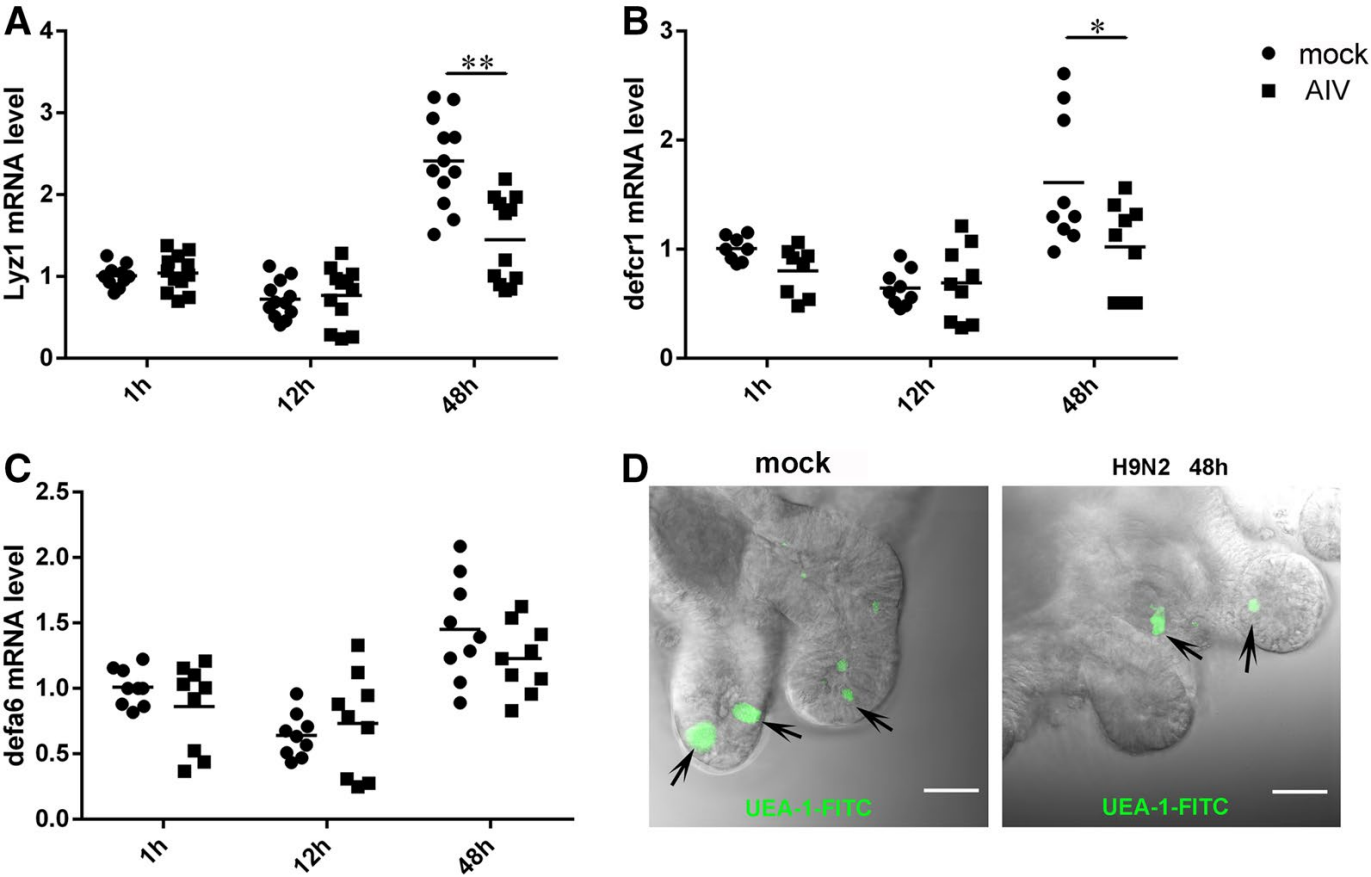
**Invasion of H9N2 virus triggers the loss of functional Paneth cells. A**–**C** RT-qPCR analysis of the expression of marker genes in Paneth cells [Lyz1 (**A**), defcr1 (**B**), and defa6 (**C**)] at 1, 12 and 48 h. The results are representative of data from duplicate three independent experiments. Each data bar shown represents mean values ± SE of triplicate assays. *P < 0.05; **P < 0.01. Bars: 30 μm.

Paneth cells synthesize and secrete antimicrobial peptides to guarantee the mucosal homeostatic balance, as well as secreting EGF, TGF-α, Wnt3 and the Notch ligand Dll4, which are essential for the maintenance of ISC [16, 17]. We further detected the changes of Paneth cells after infection with H9N2 virus. Expression of Lyz1 (Figure 4A) and defcr1 (Figure 4B) markers of Paneth cells [12, 13], were significantly reduced in H9N2 infected intestinal organoids at 48 hpi with there being no difference in Defa6 (Figure 4C) relative to mock infected cells. The mRNA transcript levels of three genes of Paneth cells were stable at 1 and 12 hpi, which may be attributed to low level virus titers in organoids as observed in Figure 2. This phenomenon observed in cultured Paneth cells in organoids was similar to observations of analogous cells made in vivo [18].

### Effects of H9N2 virus invasion on intestinal stem cell niches

**Figure 5:**
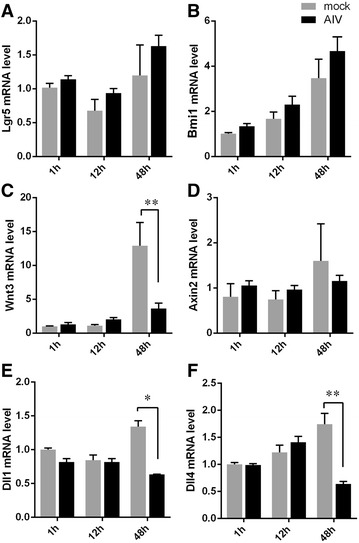
**Invasion of H9N2 virus affects ISC and Wnt signaling pathways. A**–**D** RT-qPCR analysis of the relative expression of marker genes in ISC (Lgr5, Bmi1) and Wnt signal pathway (wnt3 and Axin2) at 1, 12 and 48 h. **E**, **F** RT-qPCR determined relative mRNA expression of Notch pathway genes (Dll1 and Dll4). The results are representative of data from three independent experiments. Each data bar shown represents mean values ± SE of triplicate assays. *P < 0.05; **P < 0.01.

Intestinal homeostasis, which is regulated by proliferation and differentiation of ISC, is important for the integrity of the intestinal mucosa [19]. H9N2 virus invasion did not significantly affect the mRNA expressions of Lgr5 (Figure 5A) and Bmi1 (Figure 5B) in intestinal organoids at 48 hpi. However, infection led to organoids shrinking gradually (Figure 3A). The Wnt/β-catenin signal pathway controls epithelial proliferation, intestinal homeostasis, and ISC maintenance, while Notch signaling is in the regulation of both progenitor cell proliferation and cellular differentiation in the intestine [20, 21]. Although the mRNA transcript levels of Wnt3 (Figure 5C) and Axin2 (Figure 5D) in mouse intestinal organoids were not significantly altered after infection with H9N2 virus for 1 or 12 h, the expression level of Wnt3 was significantly reduced at 48 h (Figure 5C). Moreover, the mRNA expression levels of Dll1 (Figure 5E) and Dll4 (Figure 5F) were also markedly reduced at 48 hpi.

### Statistical analysis

Data are expressed as the mean ± SE of the mean and one-way analysis of variance (ANOVA) was used. Differences were considered significant at *P < 0.05 and **P < 0.01.

## Discussion

Intestinal organoids that consist of intestinal stem cells can develop to study intestinal viruses and crypts and represents a promising model for intestinal research [8, 22]. Intestinal organoids now have been widely used for studies of intestinal inflammation and intestinal stem cells [23, 24]. Most recently, induced human intestinal organoids (iHIOs) have been used as an intestinal model for rotavirus invasion, which demonstrated that both laboratory and clinical rotavirus isolates can replicate not only in epithelial cells but also in mesenchymal cell populations of the iHIO [25]. The unexpected finding of the infection in mesenchymal cells highlights the promise of using organoids to reveal new questions that have not been previously recognized in intestinal cell models. However, iHIO are induced by human embryonic stem cells or induced by pluripotent stem cells [26]. Our study is the first report to use mouse intestinal organoids from intestinal stem cells to infect with virus.

H9N2 virus can survive and replicate in the intestine of waterfowls and is spread through fecal matter, which is the main reason for the global epidemic potential of AIV [27, 28]. Unlike traditional intestinal cell models, such as Caco-2 cells, mouse intestinal organoids can bud and differentiate into intestine-like tissues with crypts and villi, which represent an ideal model for further studies of AIV infection. H9N2 virus could be detected in mouse intestinal organoids from 1 h post-infection, and the HA, NA, NP and PB1 mRNA transcript levels peaked at 48 h post-infection. H9N2 virus structural protein, HA, and non-structural protein, NP, could also be detected by immunofluorescence at 48 hpi. Together, these findings demonstrated that H9N2 could infect intestinal organoids. The replication peak was also consistent with morphological damage, reduced EdU staining cells and increased apoptosis cells at 48 hpi. This phenomenon may occur because gut tissues express sialic acid (SA) receptors with α2,3 linkages, which are preferentially used by avian influenza viruses, as has been shown in previous studies [5, 6]. Moreover, the levels of SA-α2,3-Gal receptor expression gradually increased from the ileum to the rectum, and these receptors were only detected in the basal layer of the small intestine, which results in direct infection by AIV H5N1 followed by replication in human gut tissues [29]. This expression of SA receptors in the basal layer could explain the invasion of AIV H9N2 into organoids from the basal side [29]. This bi-directional infection of influenza H1N1 and H5N1 viruses has also been demonstrated in alveolar epithelial cells from both apical and basolateral surfaces of the epithelium [30].

H9N2 virus induced more severe apoptosis in a human intestinal epithelial cell line, HT-29 [5]. Furthermore, AIV infection in the intestinal tract can result in severe destruction of the mucosa, which was characterized by the loss of epithelial cells and crypt distortion by histological examination [29]. This phenomenon in intestinal cells could also be observed in the intestinal organoid model. The intestinal organoids that contain intestinal stem cells can differentiate into intestinal villi that contain all types of epithelial cells, which supports the utility of organoids for studies of interactions between virus and intestine cells. The Wnt and Notch signaling pathways are the two most important regulators of ISC proliferation and differentiation [20, 31]. We found that after infection with H9N2 virus, RT-qPCR measurements of the relative mRNA transcript level of Wnt3 was significantly reduced at 48 hpi. Similar findings were also observed for the Notch pathway genes (Dll1 and Dll4). These data indicate that H9N2 virus infection could impair ISC proliferation and differentiation, which could also explain the damaged organoids observed by morphological assessments.

Paneth cells (PC) are highly specialized secretory cells located at the base of crypts in the small intestine [16]. PC play a key role by releasing granules that contain antimicrobial proteins, such as lysozyme and α-defensins or cryptdins, to protect against invasion by intestinal pathogens [12]. In addition to these antimicrobial functions, PC are a component of the intestinal stem cell niche. Paneth cells express EGF, TGF-α, Wnt3 and the Notch ligand Dll4, which are all essential signals for ISC maintenance in culture [16]. A co-culture of sorted ISC with Paneth cells markedly improves organoid formation [17]. In this present study, expression of the Paneth cell marker genes (lyz1 and defcr1) were significantly reduced at 48 hpi post-infection with H9N2 virus compared with the control group. These data imply that Paneth cells were damaged by virus invasion, which also resulted in an imbalance of ISC niches.

In conclusion, we found that H9N2 virus could invade mouse intestinal organoids using a three-dimensional intestinal model with crypts and villi. H9N2 virus invasion also resulted in reduced levels of lyz1 and defcr1 expressions, and affected the Wnt and Notch pathways to influence ISC proliferation and differentiation. Moreover, these findings demonstrate that the H9N2-infected organoid culture system represents a novel experimental model suitable for studying host–virus interactions.
